# The Use of Pathotype Data for the Selection and Development of Barley Lines with Useful Resistance to Scald

**DOI:** 10.3390/plants11192501

**Published:** 2022-09-24

**Authors:** Hugh Wallwork, Mark Butt, Milica Grcic, Tara Garrard

**Affiliations:** 1 South Australian Research and Development Institute, Adelaide, SA 5064, Australia; 2 School of Agriculture Food and Wine, The University of Adelaide, Adelaide, SA 5064, Australia

**Keywords:** barley germplasm, scald, genetic resistance, pathotype variation, introgression lines

## Abstract

Resistance in barley to scald caused by *Rhynchosporium commune* is readily overcome as virulent pathotypes in the pathogen population are selectively favoured over less virulent pathotypes. Diverse sources of resistance amongst host accessions have been found upon screening a wide array of accessions from around the world. Deciding which of these is of greatest value, and which are different from each other, takes a much greater investment of time and effort. This paper reports on the use of seedling screening techniques using 262 individual scald isolates collected from around Australia, to identify the most useful resistance sources from amongst 30 previously selected. No resistance source was effective against all isolates, but some such as Pamunkey, CI8618, CI4364 and ICARDA 4 were shown to have resistance to most isolates, whilst others were much less useful. Some of the most effective donors were shown to likely have more than one gene involved. The value of gene pyramids is discussed, as are the advantages and pitfalls of transferring the resistances from poorly adapted genetic backgrounds into better-adapted breeding lines so that they can more readily be used by breeding programs. This is a work in progress and the introgressed resistances being developed are available to all.

## 1. Introduction

Scald of barley, caused by *Rhynchosporium commune* (previously *Rhynchosporium secalis*), is a damaging pathogen of barley across the world where barley is cultivated, but particularly in higher rainfall areas [1]. No teleomorph has yet been identified for *R. commune* but a study [2] using internal transcribed spacer (ITS) region DNA sequences has suggested that the fungus is closely related to the eyespot fungus *Oculimacula yallundae*. Following initial infection, either by conidia or the as-yet undetected airborne ascospores from a sexual ascus, the pathogen is locally dispersed by rain-splash of asexual conidia within the crop canopy [3]. Besides barley, the fungus is also hosted by barley grass (*Hordeum* spp.) [4].

Pathotype studies from around the world have shown a high degree of variation in virulence in the pathogen population [5,6,7,8,9,10,11]. Ali [12] showed that scald isolates from barley grass (*Hordeum leporinum*) are highly variable for virulence and studies on barley grass populations have also shown that the grass population is also highly variable for resistance which would help to select for complex virulence patterns in the fungus [13]. Genetic studies of the pathogen [14,15] have also shown a high level of diversity on a local scale.

A study of variation in the scald population using AFLP analysis of scald hotspots in South Australia [6] has also served to illustrate how variable the pathogen can be in a single field.

Resistance to scald is controlled both by major genes that provide high levels of resistance at all growth stages (seedling resistance) and by minor genes that provide partial levels of adult plant resistance (APR) [16].

The high degree of variation in virulence in the pathogen population explains why the use of host resistance to protect crops can be problematic and why major gene resistances deployed by plant breeders have rapidly led to the detection of virulence for that gene in the pathogen population [17,18]. However, despite the high degree of variation in virulence, it is apparent from observation of variety trials in Southern Australia that most current varieties grown in the southern region of Australia possess some degree of useful resistance when compared to highly susceptible check plots. This resistance is mediated by minor genes and/or by major genes where virulence for that gene in the population is at a low level. The level of resistance observed depends on the season, location, and virulence in the local pathogen population and presumably is strongly influenced by which varieties had been grown in the area in previous years. It is hypothesised that by increasing the complexity of resistance within and between varieties, the efficacy of these resistances will be enhanced as selection for virulence in the pathogen population will be more multidirectional. Modeling of barley variety mixtures for pathogen resistance [19] provides support for the value of deploying greater complexity of resistance in the host although the use of barley mixtures per se is not a favoured option for the malting industry.

To provide the barley industry with a greater diversity of resistance in varieties, barley breeders need to be supplied with a greater range of major and minor gene resistances in agronomically adapted genotypes. This could be conducted through the pre-breeding accumulation of more minor resistance genes in gene pyramids or through the identification and development of a wider range of major seedling resistance genes.

Minor resistance genes appear to be common in barley. This is apparent when most varieties are compared to highly susceptible check varieties. Internationally, many studies have identified minor gene or quantitative trait loci (QTL) in various parents, mostly from biparental crosses [16]. However, the genetics of these resistances have yet to be thoroughly investigated and tracking of these loci in pedigrees and progeny has not been conducted to any great degree.

The published literature on seedling resistance genes in differential varieties has led to contradictory and often inconclusive information [20,21]. The reason for this has been largely due to the variable nature of the pathogen isolates or populations used and because of the sensitivity of the host-pathogen interaction to environmental variables. Wallwork and Grcic [22] reported on improved screening seedling screening techniques to reduce this variation and provide greater consistency in the phenotypic detection of specific seedling resistance genes.

The major gene *Rrs1* is found in several widely grown Australian varieties, commencing with Hindmarsh, derived from parent Dash with a favoured semi-dwarf gene. Field observations and pathotype analyses (this paper) show that the *Rrs1* gene provides very strong resistance in many situations, although where virulence is present these varieties are shown to be susceptible or very susceptible. The major gene *Rrs2* was present in several Australian varieties derived from Triumph, commencing with Franklin, and virulence to this gene became and remains quite widespread, although the gene is currently absent in most commercially grown crops. An unnamed major gene was deployed in the South Australian variety Skiff and in subsequently released related varieties. Another major gene known to have been present in Australian germplasm is the *Rh4* gene introduced via the variety Chieftain and present in the variety Navigator as well as the scald differentials Osiris and La Mesita [22].

A significant problem with identifying and using major genes is that many resistance donors have multiple undesirable agronomic traits as well as susceptibility to other diseases and/or poor-quality traits. This discourages breeders from using these resistance genes, especially when it is known that the resistance will not be durable or even whether the resistance has useful efficacy against many of the populations of the pathogen currently in the field. A range of major gene-resistance sources were identified by Wallwork and Grcic [22] but almost all these sources are very poorly adapted for cultivation and commercial use in modern agriculture.

Abbott et al. [23] developed adapted barley lines with resistance from *Hordeum spontaneum* by backcrossing the resistance into the Australian cultivar Clipper. A range of the Clipper backcross lines were used by the southern Australian breeding programs and one, AB6, is in the pedigree of the varieties Tantangara (AB6/Skiff) and Lockyer (Tantangara/VB9104). However, the AB6 resistance, which is mediated by the *Rrs13* locus identified on 6H [24], along with other sources of resistance developed from *Hordeum spontaneum* have shown susceptibility to most isolates when tested by Wallwork & Grcic [22].

This paper describes work where we have sought to reduce many of the problems associated with utilising major resistance genes. Firstly, we have identified sources of seedling resistance that appear to be different from each other based on the use of pathotype data. We have then identified those that appear to be most useful in the context of providing diverse resistance to a large proportion of isolates of the pathogen collected across Southern and Western Australia. Finally, we are in the process of transferring these resistances into a common well-adapted barley breeding line WI3580 with a reasonable grain quality profile and with useful resistance to other pathogens. These derived lines are now becoming available for use by breeding programmes.

## 2. Results

Seedling reactions of all scald isolates were assessed against a collection of scald resistance donors and susceptible check barley breeding lines WI3284 and WI3580. In Table 1 the data has been sorted so that isolates collected from across Australia are grouped into the year of collection of the isolates. Each combined score was then colour-coded to enable easier viewing with green showing a mostly resistant reaction for each barley line within each year: blue is used for intermediate levels of resistance and yellow is used for a mostly susceptible reaction. Full susceptibility is shown in orange. In Table 2, the same original data are sorted so that isolates collected across years from each state are grouped together. The same colour coding is used to help illustrate the overall efficacy of a resistance donor across years in each state.

The results in Table 1 and Table 2 show large differences in the number of resistant interactions shown by different donors from the 262 separate isolate tests conducted over 8 years. Surprisingly, both *Rrs1* in Turk and, to a lesser extent, *Rrs2* in Atlas has been providing useful levels of scald resistance. Atlas 46, which combines both *Rrs1* and *Rrs2*, shows this combination to have been particularly effective across all years. *Rrs*1 does appear to have been less effective in NSW and more effective in Tasmania.

Another gene *Rh4* present in Chieftain but not deployed to any great degree in Australia appears to have been providing little protection on its own. In combination with one or more other resistance genes in Osiris; however, this resistance has been performed very effectively, although to a lesser degree in Tasmania.

A major resistance gene is present in the variety Westminster and has been widely deployed in Australia, but the location of the gene is unknown. However, in Table 2 the resistance was largely ineffective to isolates collected from Tasmania and Victoria. In Western Australia and, to a lesser extent NSW, the Westminster resistance has shown promise.

The other gene deployed in Australian cultivars *Rrs*_Skiff_, mostly in the variety Skiff, also shows a useful level of efficacy, although considerably less so in South Australia.

When compared to these already deployed resistances, it is notable that the lines IBON 05-2, BVDV-030, and Union Beardless have shown very poor efficacy across Australia. In contrast, the resistance donors Pamunkey, CI8618 and ICARDA 4 have shown very effective resistance across years and seasons. CI4364 has also shown similar effectiveness except for 2021 isolates where a donor derivative was used for the tests in place of CI4364. It is possible that the result is due to a mixed seed, and this will be checked. Similar susceptible results in 2021 were observed with the replacement lines for the resistances in ICARDA SN3-26 and Ethiopia 5.

Of the donor resistance sources tested for only six years, Yangsimai 3, Pennco, PC249 and Wiebe GA97-9 (Ethiopia 4) all show good promise as useful donors of resistance.

The barley lines CI8288, Ethiopia 107, Orge 618, Quina, Gatillo Bar, WA9718, Ethiopia 5, Ethiopia 317, and Zavilla all warrant further development for use by breeders.

The pathotype data for 2021 where the donors Turk, ICARDA 4 and Pamunkey have been replaced by Spartacus and two WI3580 derivatives, respectively, show a loss of resistance when compared to ICARDA 4 and Pamunkey in previous years.

A comparison of the efficacy of different resistances across states indicates remarkable consistency overall. The mean level of resistance by 32 barley lines to 262 isolates did not change from one state to another with the possible exception of Tasmania where the virulence of the pathotypes was slightly greater. At just 12, however, the total number of isolates collected from Tasmania was a lot lower than from the other states, which may account for the difference.

One isolate, 1972, collected from a barley crop near Rochester in 2020 has been identified as having an exceptional level of virulence and is shown in Table 3. This is the only isolate detected that shows virulence on Pamunkey. It notably also has virulence on all other resistance sources other than CI8618 and Ethiopia 317. It also has just partial virulence on Skiff.

## 3. Discussion

The pathotype results presented here provide a comprehensive view of the virulence of the scald population across Australia over an eight-year period. Resistances challenged include genes that have been deployed in Australian cultivars as well as a range of other resistances that are presumed to have been absent in commercial varieties. That none of the resistances have held up to all isolates shows that variation in the pathogen population is high and probably capable of evolving to overcome any variety that is likely to be developed based on major seedling resistance genes. Indeed isolate 1972 has shown virulence on all but three of the thirty different resistance donors investigated here and two of those donors have shown susceptibility to other isolates (Table 3).

Virulence on the third donor, Ethiopia 317, has only been tested with 38 isolates so far and is not expected to be any more durable than other sources. The very similar level of pathogen diversity across the different geographic regions, including virulence for genes not thought to be present in recent or historical commercial cultivars, is a further indication of the overall diversity of the pathogen in the field. This could be a consequence of genetic diversity in resistance in the barley grass population across Australia ensuring selection is constantly made for a diversity of resistance in the pathogen.

The use of gene combinations, and in particular diverse gene combinations in different varieties, is however likely to present a powerful tool for delaying and minimizing damage from scald. A strategy that more broadly is promoted in cereal foliar disease resistance to sustain durable resistance [18]. This is well illustrated with the effectiveness of Atlas 46 compared to Turk and Atlas and also the effectiveness of Osiris which is considered to have at least two major seedling resistance genes including *Rh4* that is present in Chieftain.

Pamunkey which has demonstrated mostly very effective resistance in these tests is also thought to contain two major seedling resistance genes against isolate 6 [22] on 1H and 3H based on marker studies on the population Pamunkey/WI3580 by Mather et al. (pers comm). Tests on the lines resistant to isolate 6 using isolate 332a show susceptibility in about half of the lines suggesting one of the two resistance genes could be the *Rrs1* gene on 3H. Additionally, the virulence pattern on the WI3580-derived line suggests that Pamunkey carries the *Rrs1* gene. Whilst this gene alone appears to provide useful benefits, the addition of a second gene on 1H is observed to confer a greater advantage. The virulence of these genetics has not been tested or published internationally.

The resistance in ICARDA 4, also likely based on *Rrs1*, is probably bolstered by a second gene that is absent from the WI3580 derived line. ICARDA 4 has also been reported to have held up resistance to Ethiopian and Moroccan isolates [25].

On close inspection, it is apparent that ICARDA 4 and Pamunkey are reacting similarly to the 41 isolates in the tests. This suggests they both have the same *Rrs1* resistance and that the selected derived lines have lost a second resistance gene present in each of the donor parents that gave them a different and more effective resistance profile in earlier tests. New selections will therefore be made from the populations already developed to identify alternative lines that carry the full donor resistances. This can be conducted using one of the isolates collected in previous years that showed different reactions on Turk compared to ICARDA 4 and Pamunkey. Comparing pathotype reactions between ICARDA 4 and Pamunkey reveal several isolates that respond differently to the two donors suggesting that if both varieties do carry *Rrs1*, then at least the second gene in each variety is different and therefore resistance from both donors is worth pursuing.

Despite not having been deployed in any commercial varieties, the sources IBON 05-2, BVDV-030 and Union Beardless have not shown sustained resistance, despite showing promise with initial resistance testing against limited isolates.

The results obtained from including lines derived from the transfer of donor resistance into the breeding lines WI3580 were mixed. For Ethiopia 107, Skiff, Orge 618, Yangsimai 3 and Pennco the transfer of resistance into a well-adapted background was successful. The derived lines from CI8618, Ethiopia 317, Quina, Gatillo Bar, showed a reduction in resistance, perhaps due to the loss of a minor gene, but otherwise, the transfer was also successful.

The line CI8618 contains one major gene as identified by Starling in 1971 [26]. A South African study with isolates collected from the Western Cape region found that 90% of isolates tested contained virulence on this line [27]. Whereas an Australian study in the 1970′s found only 6% of Southern Australian isolates tested had virulence on CI8618 [5]. Considering virulence on the line exists both internationally and in Australia the gene should be deployed in combination with one or more other genes.

Where the donors appeared to have multiple genes, e.g., Pamunkey and ICARDA 4, then it was observed that problems arose with a loss of one of the genes during the selection process. This is particularly likely to occur when one of the genes is very effective, as *Rrs*1 is, so the effect of the second gene is hard to visualise. To avoid that, a new screening will be carried-out with isolates that have been identified to differentiate between the original donor and a line known to only carry one of the genes, in this case, *Rrs*1. Better still would be to develop and use linked markers that can identify the presence of each of the genes. These resources were not, however, available for this work in recent years. In three cases, CI4364, Ethiopia 5 and ICARDA SN3-26, the derived line is seen to have lost efficacy. This could be due to residual heterozygosity or seed mixing in the selection and multiplication processes. This can be corrected with a return to and multiplication of the original selections.

Development of these resistant sources into the Australian adapted WI3580 line provides highly valuable advancements in barley scald resistance to the Australian industry. This paper illustrates the diversity of resistance sources available to breeders and an understanding of their performance nationally over an eight-year period. From this work, and over the next couple of years, lines are and will become available for use by breeding programs, enabling them to readily incorporate a more diverse range of resistances into new varieties.

Obtaining the chromosomal location of each of the genes in each of the donors will be an important factor in the successful pyramiding of these major genes. Firstly, it will ensure that each of the genes can be pyramided and are not located at the same position on the same chromosome, secondly, it will greatly assist in the rapid selection of lines with the combined genes. Thirdly, it will provide breeders who wish to use these gene pyramids with the means to do so with the greatest efficiency.

## 4. Materials and Methods

A collection of resistance donors was made based on the reaction of many barley accessions to four differential isolates as described in Wallwork and Grcic [22]. A collection of donors that showed a different pattern of resistance to multiple isolates when compared to other donors were selected for crossing and development of new scald differentials. These are listed in Table 4.

The barley varieties Turk (*Rrs1*), Atlas (*Rrs2*), Atlas 46 (*Rrs1* + *Rrs2*), Chieftain (*Rh4*) and Osiris (*Rh4* + *Rh?*) were included in seedling pathotype tests as checks. In 2021, Spartacus^®^ replaced Turk as a better adapted *Rrs1* differential. Additionally, included as seedling susceptible checks were the barley breeding line WI3284 and, in two years, WI3580. WI3580 was identified by the University of Adelaide barley breeding program as a good recipient for scald resistance, with good agronomic and quality characteristics whilst being susceptible to scald but having useful resistance to other foliar pathogens.

Many of the resistance sources in Table 4 were obtained after the preliminary screening of large numbers of accessions for multiple traits by Christy Grimes and Tefera Angessa in the University of Western Australia. Lines showing some promise for scald resistance were forwarded to SARDI for more detailed evaluation. The lines Orge 618, Zavilla and Gatillo Bar were provided by Hugo Vivar from the ICARDA barley breeding program at CIMMYT.

ICARDA 4 was identified in a study investigating 26 sources of scald resistance provided by ICARDA in 1992 [20]. The resistance had been shown to carry resistance on chromosome 3H being 99 percent linked to Bmag603 [28] but was resistant to more isolates than *Rrs1* also located close to this marker.

Once promising donor lines were identified, they were in most cases crossed to WI3580. From these crosses, either a doubled haploid population was generated or else a single-seed descent population was developed to the F5 generation. These populations were then screened as seedlings and in the field to identify lines that carried the scald resistance of the donor but were otherwise most similar to WI3580 for other traits. The selected lines were then crossed again to WI3580 and a second population developed similarly to the first. As with the first populations, the most desirable second-generation lines were selected and then used as new differentials in the pathotype testing starting for some donors from the 2020 season with the list extended in 2021. These lines are also listed in Table 4. The pedigree of Yangsimai 3 was different in that a well-adapted doubled haploid (DH) line from a mapping population, Yangsimai 3/Hindmarsh, was selected. Hindmarsh, being a well-adapted variety, meant that one crossing of the DH line with WI3580 would be enough to obtain a well-adapted differential line replacement. Mapping studies by the University of Adelaide (Mather pers comm.) showed that the scald resistance was on 6H and the selected line carried the resistant allele at this locus. Mapping studies by Mather et al. (pers comm) on a Zavilla/WI3580 DH population, identified the seedling resistance at the same locus on 6H as Yangsimai. Pathotype data at that time indicated that the Yangsimai resistance had better efficacy than the Zavilla resistance, so the use of the latter was temporarily halted in 2019.

Development of the line HW686-39 from the donor ICARDA SN3-26 followed a different path. A first round of crossing was with an off-type of the variety Gairdner, and the second cross was a breeding line WI4385. These crosses were originally made for the purpose of utilising the resistance in this parent to spot form net blotch (*Pyrenophora teres f maculata*).

The variety Westminster was included after the regular screening of commercial varieties identified a promising seedling resistance gene in this introduced variety.

### 4.1. Collection and Storage of Isolates

Scald infected leaves were collected from commercial barley crops, barley trial plots and barley grass (*Hordeum* sp.) plants growing across Southeastern and Western Australia over 7 years. Most isolates came from quite separate locations and no more than 2 isolates came from the same location in any one year.

Leaf pieces of 9 mm^2^ were excised from lesions, surface sterilised by dipping briefly in 70% ethanol and then into 2% sodium hypochlorite for 30 s. The leaf pieces were then rinsed in sterile reverse osmosis (RO) water and dried on sterile filter paper. Pieces were then placed on 3.9% Potato Dextrose Agar (Difco) amended with 0.01 g/L streptomycin sulphate and incubated at 16 °C with a 12 h photoperiod. After 2–10 days conidia that exuded in small clear, white to pink droplets were streaked with a sterile needle onto lima bean agar amended with 0.01 g/L streptomycin sulphate (amended LBA). After 24 h at 16 °C with a 12 h photoperiod, a germinated spore observed under a stereomicroscope at 100× magnification was excised from the agar using a flattened end of a platinum wire and transferred to amended LBA. The agar plate was incubated as described above and after a colony was formed it was spread over the plate with a sterile scalpel to increase growth. After 7 days of incubation, the plate was flooded with sterile RO water and the spores were scraped into solution. For long-term storage, 1 mL of the concentrated spore solution was pipetted into 2 mL Nunc cryotubes which were then filled with a mixture of sterilised silica (100–200 mesh) and self-indicating silica gel ensuring that there were at least a few indicator beads per tube. These cryotubes were stored at −80 °C. When required, pieces of silica gel were removed from cryotubes and placed directly onto amended LBA with 1 mL sterile RO water and left at 16 °C with a 12 h photoperiod for 7–10 days. 3–5 mL sterile RO water was then added, the culture was then scraped, and the spore solution was spread onto fresh amended LBA plates. These were left to grow at 16 °C with a 12 h photoperiod for seven days to produce plates covered in spores.

### 4.2. Seedling Inoculation

Barley seeds of different varieties and accessions were sown in 80 mm square pots. Each pot had 4 seeds sown of the barley lines. Plants were grown in a controlled environment room (CER) with a 10 and 14 h photoperiod set at 15 and 13 °C, respectively, until they reached the 3 leaf stage, at which time they were ready for inoculation.

Spore solutions were prepared by scraping culture plates with 5 mL RO water which were then diluted to 1 × 10^6^ spores/mL in RO water by use of a haemocytometer. One droplet of Tween 20 was added to 100 mL of spore solution which was then sprayed onto the barley seedlings. Inoculation of seedlings placed on trolleys was carried out at a remote position in the building to minimise contamination between isolates where more than one was used at a time. Following inoculation, the seedlings were placed in plastic tents in a CER in the dark with 100% humidity at 13 °C for 24 h. The CER was then set to a 10/14 h photoperiod and humidity in the tents was maintained at 100% humidity and between 13–18 °C. The first signs of infection were observed after 10 to 12 days and resistance assessments were made 14 days after inoculation.

Testing of each variety by isolate combination used 4 seedlings per pot, with 2 replicates per test.

Seedlings were rated using a simple 4-point scale (R, MR, MS and S) reflecting a lack of variation in reaction types. R = no symptoms, MR = minor lesions only, mostly on leaf sheaths or leaf margins, MS = few and/or late-appearing susceptible leaf lesions, covering less than 50% of leaf surface area, S = many large lesions or death of seedling leaves.

### 4.3. Data Preparation and Interpretation

Reactions rated as resistant (R) or moderately resistant (MR) were counted as resistant. Reactions rated as susceptible (S) or moderately susceptible (MS) were counted as susceptible. Some lines had a mixed (MRMS) response with some seedlings showing an MR and some MS. Some changes in donors included in the tests changed over time with several being added to the tests from 2016 onwards.

The reactions were then converted to the fraction of reactions rated resistant and data were grouped by the year isolates were collected and secondly by the state isolates were collected from.

## Figures and Tables

**Table 1:** Fraction of reactions rated resistant to scald isolates collected from around Australia in the years 2014–2021.

	2014	2015	2016	2017	2018	2019	2020	2021
No. Isolates	18	27	21	33	35	49	38	41
**Turk**	0.88	0.90	0.86	0.88	0.80	0.84	0.68	0.73 *
**Atlas**	0.73	0.76	0.33	0.76	0.57	0.59	0.66	0.56
**Atlas 46**	0.92	1.00	0.90	0.97	1.00	0.98	0.92	0.90
**Chieftain**	0.46	0.29	0.33	0.18	0.34	0.41	0.47	0.32
**Osiris**	1.00	0.95	0.86	1.00	0.89	0.90	0.92	0.76
**Skiff**	0.69	0.38	0.57	0.79	0.54	0.78	0.74	0.59 *
**BVDV-030**	0.50	0.33	0.38	0.27	0.31	0.29	0.47	-
**C2-05-337-2**	0.58	0.52	0.81	0.52	0.57	0.55	-	-
**CI 8618**	1.00	1.00	1.00	1.00	0.97	0.98	0.92	0.83 *
**CI4364**	1.00	1.00	1.00	1.00	0.94	0.88	0.95	0.29 *
**CI8288**	1.00	0.95	0.90	0.94	0.80	0.73	0.84	0.78
**Ethiopia 107**	0.73	0.67	0.71	0.88	0.66	0.88	0.74 *	0.63 *
**IBON 05-2**	0.54	0.24	0.33	0.12	0.11	0.10	-	-
**ICARDA 4**	1.00	1.00	0.95	1.00	1.00	0.90	0.97	0.71 *
**Jet**	0.69	0.38	0.57	0.52	0.43	0.43	0.74	0.27 *
**Orge 618**	0.85	0.81	0.86	0.82	0.83	0.80	0.61	0.63 *
**Pamunkey**	1.00	1.00	1.00	1.00	1.00	1.00	0.97	0.71 *
**Quina**	0.92	0.95	0.86	1.00	0.83	0.76	0.66 *	0.49 *
**ICARDA SN3-26**	-	-	-	-	0.94	0.96	0.82	0.37 *
**Union Beardless**	0.46	0.48	0.38	0.33	0.34	0.29	0.32 *	0.12 *
**WA9718**	0.73	0.71	0.76	0.85	0.71	0.90	-	-
**Zavilla**	0.77	0.90	0.67	0.91	0.71	0.69	-	-
**Yangsimai**	-	-	0.90	1.00	0.91	0.84	0.74 *	0.85 *
**Gatillo Bar**	-	-	-	-	0.91	0.92	0.95	0.71 *
**Westminster**	-	-	0.38	0.21	0.86	0.37	0.55	0.32
**WIEBE GA 97-9**	-	-	0.90	0.97	0.97	0.86	0.97	0.88
**Ethiopia 5**	-	-	0.71	0.94	0.77	0.73	0.92	0.27 *
**Pennco**	-	-	1.00	1.00	0.94	1.00	0.89	0.83 *
**PC249**	-	-	0.81	0.94	0.89	0.86	0.95	0.88
**Ethiopia 317**	-	-	-	-	-	-	1.00	0.71 *
**WI3284**	0.00	0.00	0.00	0.03	0.00	0.00	0.00	-
**WI3580**	-	-	-	-	-	0.00	0.00	-

Figures with an * indicate where the original donor was replaced with a WI3580 derivative. Green indicates mostly resistant reactions, yellow and orange indicate mostly susceptible reactions, and blue indicates intermediate reactions.

**Table 2:** Fraction of reactions rated resistant to scald isolates collected in different states between the years 2014–2021. The resistance for each donor includes data in 2020 and 2021 from the derived donor as shown in this table.

Donor Line	SA	Isolates	Years	Vic	Isolates	Years	WA	Isolates	Years	NSW	Isolates	Years	Tas	Isolates	Years
Turk	0.85	127	8	0.75	60	7	0.90	42	7	0.50	21	6	1.00	12	4
Atlas	0.78	127	8	0.58	60	7	0.29	42	7	0.54	21	6	0.50	12	4
Atlas 46	0.99	127	8	0.90	60	7	1.00	42	7	0.77	21	6	1.00	12	4
Chieftain	0.25	127	8	0.27	60	7	0.67	42	7	0.73	21	6	0.00	12	4
Osiris	0.90	127	8	0.90	60	7	1.00	42	7	0.88	21	6	0.67	12	4
Skiff	0.45	112	7	0.87	53	6	0.88	42	7	0.73	15	5	0.75	9	3
BVDV-030	0.28	92	6	0.34	47	5	0.38	32	6	0.90	10	4	0.11	7	2
C2-05-337-2	0.74	127	8	0.45	60	7	0.33	27	5	0.73	21	6	0.00	12	4
CI 8618	0.95	127	8	0.98	60	7	0.90	42	7	1.00	21	6	0.92	12	4
CI4364	0.87	127	8	0.88	60	7	0.79	42	7	0.81	21	6	0.83	12	4
CI8288	0.87	127	8	0.85	60	7	0.76	42	7	0.96	21	6	0.67	12	4
Ethiopia 107	0.57	127	8	0.90	60	7	1.00	42	7	0.77	21	6	0.83	12	4
IBON 05-2	0.17	112	6	0.15	47	5	0.22	27	5	0.73	15	5	0.00	7	2
ICARDA 4	0.96	127	8	0.88	60	7	0.93	42	7	0.85	21	6	1.00	12	4
Jet	0.46	127	8	0.47	60	7	0.62	42	7	0.69	21	6	0.08	12	4
Orge618	0.81	127	8	0.70	60	7	0.86	42	7	0.42	21	6	0.92	12	4
Pamunkey	0.98	127	8	0.93	60	7	0.93	42	7	0.85	21	6	1.00	12	4
Quina	0.79	127	8	0.73	60	7	0.93	42	7	0.85	21	6	0.25	12	4
ICARDA SN3-26	0.93	82	5	0.72	32	4	0.86	29	4	0.46	13	3	0.80	10	3
Union Beardless	0.33	127	8	0.38	60	7	0.19	42	7	0.46	21	6	0.00	12	4
WA9718	0.65	92	6	1.00	47	5	1.00	27	5	0.53	10	4	1.00	7	2
Zavilla	0.73	92	6	0.85	40	5	0.85	27	5	0.67	10	4	0.57	7	2
Yangsimai 3	0.90	101	6	0.80	54	6	0.94	36	5	0.57	14	4	1.00	12	4
Gatillo Bar	0.89	82	6	0.78	32	4	0.97	29	4	0.85	13	3	0.80	10	3
Westminster	0.44	101	6	0.30	54	6	0.78	36	5	0.64	14	4	0.00	12	4
WIEBE GA 97-9	0.94	101	6	0.85	54	6	0.97	36	5	0.86	14	4	1.00	12	4
Ethiopia 5	0.74	101	6	0.69	54	6	0.75	36	5	0.57	14	4	0.67	12	4
Pennco	0.96	101	6	0.91	54	6	0.97	36	5	0.79	14	4	1.00	12	4
PC249	0.87	101	6	0.87	54	6	0.97	36	5	1.00	14	4	0.75	12	4
Ethiopia 317	0.80	35	2	0.92	13	2	0.80	15	2	1.00	11	2	0.80	7	2
WI3284	0.01	112	7	0.02	53	7	0.00	32	6	0.00	15	5	0.00	9	3
WI3580	0.00	40	2	0.00	21	2	0.00	12	2	0.00	7	2	0.00	7	2
**Mean**	**0.68**			**0.68**			**0.73**			**0.69**			**0.59**		

Green indicates mostly resistant reactions, yellow and orange indicate mostly susceptible reactions and blue indicates intermediate reactions.

**Table 3:** Pathotype results of isolate 1972 collected in a barley crop near Rochester, Victoria in 2020.

Barley Line	Scald Rating
**Turk**	**SMS**
**Atlas**	**S**
**Atlas 46**	**S**
**Chieftain**	**S**
**Osiris**	**S**
**Skiff**	**MRMS**
**BVDV-030**	**S**
**CI8618**	**MR**
**CI4364**	**MS**
**CI8288**	**MS**
**HW715DH-129** (*Ethiopia 107*)	**SMS**
**ICARDA 4**	**S**
**Jet**	**S**
**Orge 618**	**S**
**Pamunkey**	**S**
**HW775DH-023** (*Quina*)	**S**
**ICARDA SN3-26**	**S**
**HW712DH-040** (*Union Beardless*)	**S**
**HW747DH-039** (*Yangsimai 3*)	**S**
**Gatillo Bar**	**SMS**
**Westminster**	**S**
**WIEBE GA 97-9**	**MS**
**Ethiopia 5**	**MS**
**Pennco**	**S**
**PC249**	**SMS**
**Ethiopia 317**	**MR**
**WI3284**	**S**
**WI3580**	**S**

**Table 4:** Sources of scald resistance, alternative identifiers and other information relating to them, SARDI = South Australian Research and Development Institute; AGG = Australian Grains GeneBank; ICARDA—International Centre for Agricultural Research in the Dry Areas; UWA = University of Western Australia; USDA = United States Department of Agriculture.

Donor	Source/Accession Numbers	Notes
Turk	SARDI reselected, AGG495286	Differential for *Rrs1*
Atlas	SARDI reselected, AGG495287	Differential for *Rrs2*
Atlas 46	SARDI reselected, AGG495288	Differential for *Rrs1* + *Rrs2*
Osiris	SARDI reselected, AGG495289	Differential for *Rh4* + ?
Chieftain	AGG407452	UK variety used as differential for *Rh4*
Westminster	AGG422888	UK variety
Union Beardless	AGG402167, WA9621	Old Argentinian variety, 6 row
Ethiopia 107	AGG422434, CI3915-1	6 row, ex Ken Sato Okayama, waterlogging
Pamunkey	USDA PI583865, AGG422518	6 row, winter feed variety ex USA
Quina	ICARDA, AGG406867	
WA9718	AGG495294	
BVDV-30	ICARDA, AGG495295	ICARDA line with BYDV resistance
C2-05-337-2	UWA, AGG495297	Hooded barley
IBON 05-2	ICARDA, AGG495296	ICARDA line
Yangsimai 3	China, AGG411530	Good *P. teres f maculata* resistance
CI8618	AGG403502	
CI4364	Von Bothmer, AGG495176	6 row
Jet	AGG495290, CI967	Black, hulless grain from Ethiopia
ICARDA 4	ICARDA BSCGP-99, AGG495291	Arar/Lignee 527, ICB85-0625-6AP-0AP-29APH-0AP, 6 row
ICARDA SN3-26	ICARDA, AGG495292	Abeto’S’//Gloria’S’/Come’S’/3/Sen’S’, 6 row
Orge 618	CIMMYT/ICARDA, AGG495293	ICARDA, CID201269, CI12023
Gatillo Bar	CIMMYT/ICARDA, AGG408548	CID162240 GID 174815, 6 row
Zavilla	CIMMYT/ICARDA, AGG408547	6 row
Ethiopia 5	USDA, AGG400103	Black heads, very tall
Ethiopia 317	ICARDA, AGG408657	Very tall
Pennco	USDA, AGG412680, Fig041	6 row, awnless US variety
Wiebe GA97-9	USDA, AGG400102	Ethiopia 4
PC249	USDA, AGG412760, Fig111	6 row, very tall
Skiff	University of Adelaide, AGG403001	Australian variety
WI3284	University of Adelaide	Susceptible line used as check
WI3580	University of Adelaide	Susceptible line used a recipient for resistance
Spartacus	Intergrain, Australia	Australian variety carrying *Rrs1*
HW668-56	SARDI	Developed from Gatillo Bar
HW673-171	SARDI	Developed from Orge 618
HW683-1	SARDI	Developed from Jet
HW686-39	SARDI	Developed from ICARDA SN3-26
HW712DH-040	SARDI	Developed from Union Beardless
HW715DH-129	SARDI	Developed from Ethiopia 107
HW730-105	SARDI	Developed from CI4364
HW747DH-039	SARDI	Developed from Yangsimai 3
HW762-218	SARDI	Developed from Pamunkey
HW773-085	SARDI	Developed from Skiff
HW775DH-062	SARDI	Developed from Quina
HW782DH-001	SARDI	Developed from ICARDA 4
HW786-051	SARDI	Developed from Ethiopia 5
HW787-10	SARDI	Developed from Ethiopia 317
HW788-030	SARDI	Developed from Pennco
HW789-083	SARDI	Developed from CI8618

## Data Availability

Data supporting the reported results are available from the Corresponding author.

## References

[1] Abebe W. (2021). Review on Barley Scald Disease Management. Int. J. Environ. Agric. Res..

[2] Goodwin S.B. (2002). The barley scald pathogen *Rhynchosporium secalis* is closely related to the discomycetes *Tapesia* and *Pyrenopeziza*. Mycol. Res..

[3] Avrova A., Knogge W. (2012). *Rhynchosporium commune*: A persistent threat to barley cultivation. Molec. Plant Pathol..

[4] Linde C.C., Smith L.M., Peakall R. (2016). Weeds, as ancillary hosts, pose disproportionate risk for virulent pathogen transfer to crops. BMC Evol. Biol..

[5] Ali S.M., Mayfield A.H. (1976). Clare BG Pathogenicity of 203 isolates of *Rhynchosporium secalis* on 21 barley cultivars. Physiol. Plant Pathol..

[6] Williams K., Donellan S., Smyl C., Scott L., Wallwork H. (2003). Molecular variation in *Rhynchosporium secalis* isolates obtained from hotspots. Austral. Plant Pathol..

[7] Jackson L.F., Webster R.K. (1976). Race differentiation, distribution and frequency of *Rhynchosporium secalis* in California. Phytopathology.

[8] Tekauz A. (1991). Pathogenic variation in *Rhynchosporium secalis* on barley in Canada. Can. J. Plant Pathol..

[9] Goodwin S.B., Allard R.W., Hardy S.A., Webster R.K. (1992). Hierarchical structure of pathogenic variation among *Rhynchosporium secalis* populations in Idaho and Oregon. Can. J. Bot..

[10] Zhang Q.F., Webster R.K., Crandall B.A., Jackson L.F., Maroof M.A.S. (1992). Race composition and pathogenicity associations of *Rhynchosporium secalis* in California. Phytopathology.

[11] Salamati S., Tronsmo A.M. (1997). Pathogenicity of *Rhynchosporium secalis* isolates from Norway on 30 cultivars of barley. Plant Pathol..

[12] Ali S.M. (1981). Barley grass as a source of pathogenic variation in *Rhynchosporium secalis*. Aust. J. Agric. Res..

[13] Jarosz A.M., Burdon J.J. (1996). Resistance to barley scald (*Rhynchosporium secalis*) in wild barley grass (*Hordeum glaucum* and *Hordeum leporinum*) populations in South-Eastern Australia. Aust. J. Agric. Res..

[14] Linde C.C., Zala M., McDonald B.A. (2009). Molecular evidence for recent founder populations and human-mediated migration in the barley scald pathogen *Rhynchosporium secalis*. Mol. Phylogenet. Evol..

[15] McDonald B.A. (2015). How can research on pathogen population biology suggest disease management strategies? The example of barley scald (*Rhynchosporium commune*). Plant Pathol..

[16] Zhang X., Ovenden B., Milgate A. (2020). Recent insights into barley and *Rhynchosporium commune* interactions. Mol. Plant Pathol..

[17] Burdon J.J., Barrett L.G., Rebetzke G., Thrall P.H. (2014). Guiding deployment of resistance in cereals using evolutionary principles. Evol. Appl..

[18] Mundt C.C. (2014). Durable resistance: A key to sustainable management of pathogens and pests. Infect. Genet. Evol..

[19] Wolfe M.S. (1985). The current status and prospects of multiline cultivars and variety mixtures for disease resistance. Annu. Rev. Phytopathol..

[20] Boyd W.J.R., Janakiram D., Palaklang W. Quantitative response to scald (*Rhynchosporium secalis*). Proceedings of the Barley Genetics V: Fifth International Barley Genetics Symposium.

[21] Goodwin S.B., Allard R.W., Webster R.K. (1990). A nomenclature for *Rhynchosporium secalis* pathotypes. Phytopathology.

[22] Wallwork H., Grcic M. (2011). The use of differential isolates of *Rhynchosporium secalis* to identify resistance to leaf scald in barley. Austral. Plant Pathol..

[23] Abbott D.C., Brown A.H.D., Burdon J.J. (1992). Genes for scald resistance from wild barley (*Hordeum vulgare* ssp. *spontaneum*) and their linkage to isozyme markers. Euphytica.

[24] Abbott D.C., Lagudah E.S., Brown A.H.D. (1995). Identification of RFLPs flanking a scald resistance gene on barley chromosome 6. J. Heredity.

[25] Hiddar H., Rehman S., Lakew B., Pal Sing Verma R., Al-Jaboobi M., Moulakat A., Kehel Z., Filali-Maltouf A., Baum M., Amri A. (2021). Assessment and modeling using machine learning of resistance to scald (*Rhynchosporium commune*) in two specific barley genetic resources subsets. Sci. Rep..

[26] Starling T.M., Roane C.W., Chi K.R. Inheritance of reaction to *Rhynchosporium secalis* in winter barley cultivars. Proceedings of the 2nd International Barley Genetics Symposium.

[27] Robbertse B., Lennox C.L., Van Jaarsveld A.B., Crous P.W., Van Der Rijst M. (2000). Pathogenicity of the *Rhynchosporium secalis* population in the Western Cape province of South Africa. Euphytica.

[28] Genger R., Williams K.J., Raman H., Read B., Wallwork H., Burdon J.J., Brown A.H.D. (2003). Leaf scald resistance genes in *Hordeum vulgare* and *Hordeum vulgare* ssp. *spontaneum*: Parallels between cultivated and wild barley. Aust. J. Agric. Res..

